# Supplementary figures and images for: Machine learning predicts cancer subtypes and progression from blood immune signatures
Source: PLoS One. 2022 Feb 28;17(2):e0264631. doi: 10.1371/journal.pone.0264631 (PMC8884497; doi:10.1371/journal.pone.0264631)

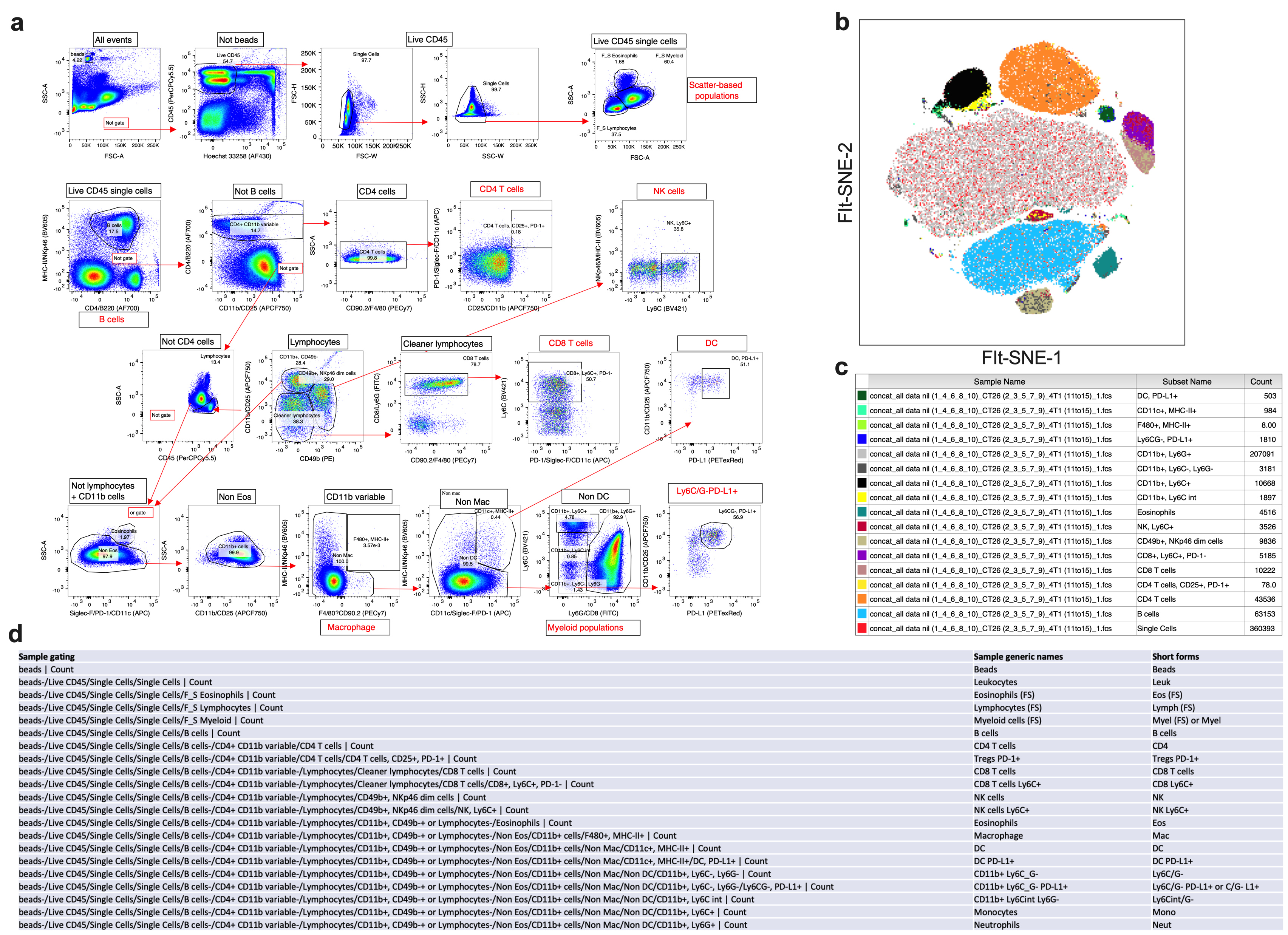

Supplement: S1 Fig — FlowJo software was used to delineate leukocyte populations using manual and boolean gates on concatenated samples with the scheme shown in (a) acting as a template for the entire study. FIt-SNE plots from concatenated live CD45+ samples, generated with default FlowJo setting, were overlayed with each manual gated population to ensure the gating scheme generated similar populations to those generated from the unsupervised approach (b), with the manual gate population identified by colour and name (c). The process was refined until the two approaches were good approximations of each other, resulting in the manual gates displayed in a. Generic and short form names of each population were then assigned based on marker expression and used throughout the manuscript (d). (TIFF) [file pone.0264631.s001.tiff]

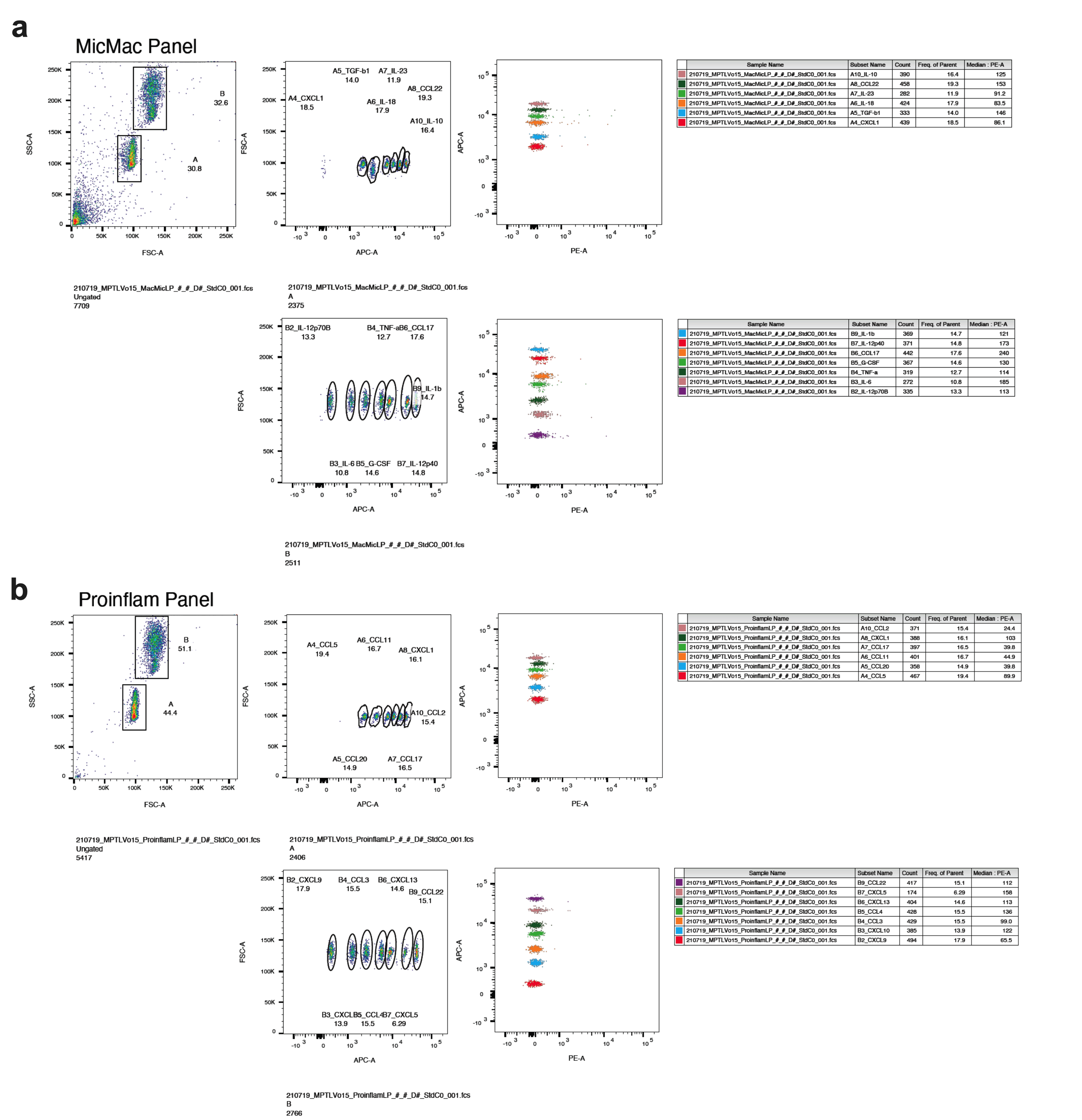

Supplement: S2 Fig — FlowJo software was used to delineate LEGENDplex bead populations using manual gates for both the Macrophage/microglial (Mac/Mic) 13-plex LEGENDplex kit (a) and the Proinflammatory (Proinflam) 13-plex LEGENDplex Kit (Biolegend) (b), which acted as a template for the entire study. (TIFF) [file pone.0264631.s002.tiff]

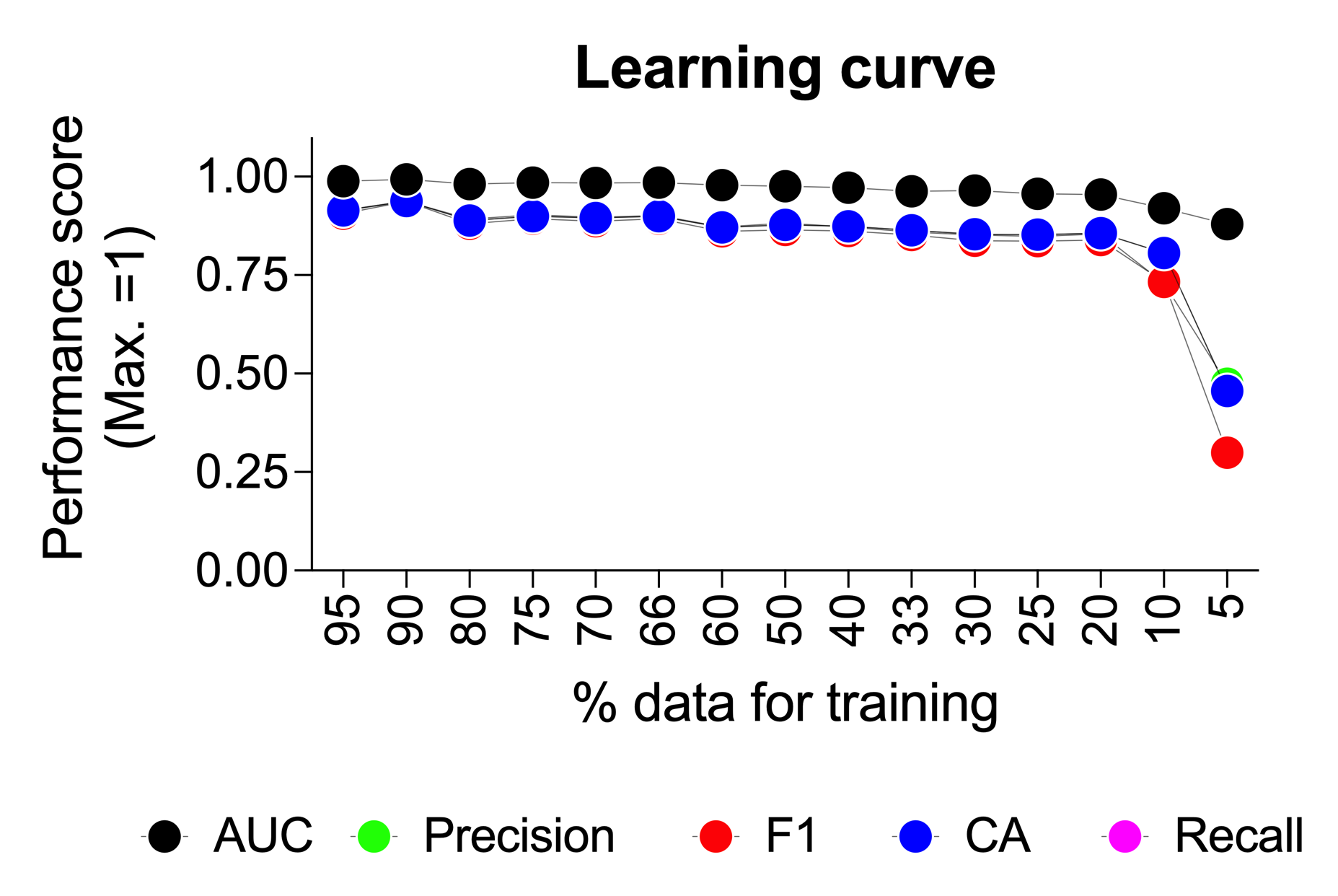

Supplement: S3 Fig — Normalised blood immune features (S4 Fig) taken from the 130 animals that had both day 7 and day 14 blood samples (Fig 1B), were used in Random Forest modelling to predict presence of tumour and tumour subtype (targets class being Nil, 4T1 and CT26). Modelling was done on a progressively smaller number of random samples and model performance assessed using cross-validation with a training set of 80% of randomly obtained data and tested on the remaining data and this repeated 100 x. Model performance was assessed by several classification indicators, including area under curve of the receiver operating characteristics (AUC; to assess separability of the classes), classification accuracy (CA; proportion of correct classification), precision (ratio of correct positive prediction to all predicted positive), recall (ratio of correct positive prediction to actual positive), and F1 score (weighted average of precision and recall) with values being from 0 to 1 (and toward the latter being the best). (TIFF) [file pone.0264631.s003.tiff]

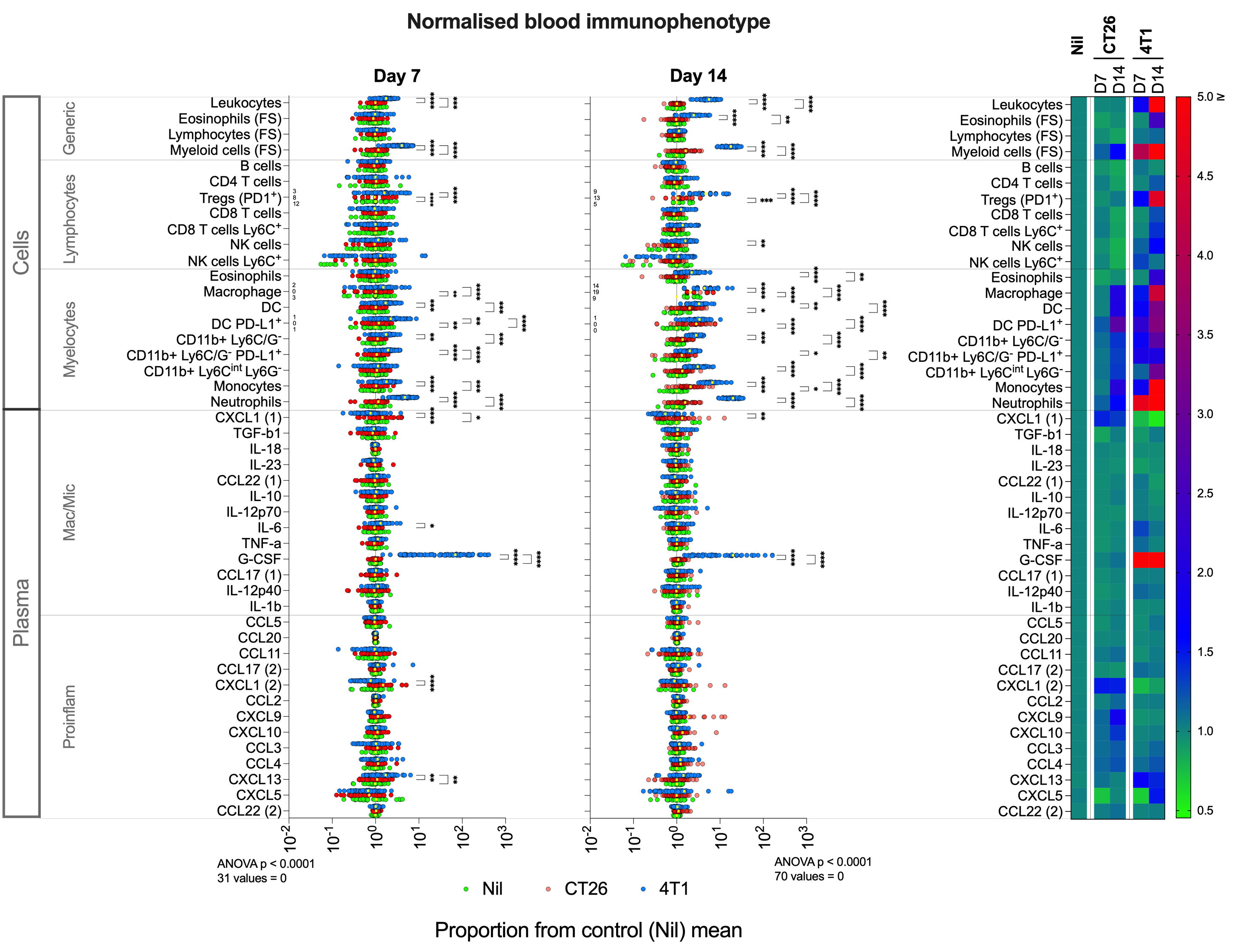

Supplement: S4 Fig — CT26 or 4T1 tumours were grown in female, BALB/c mice and blood immune phenotype determined at D7 and D14, as described in Fig 1. Animals with no tumours (Nil) were used as normal immune phenotype controls. A total of 180 animals were included in the study, and animals divided into the groups indicated in Fig 1B. A 20 μl of blood sample from each animal at each time point was phenotyped for leukocyte populations and plasma analytes (Fig 1). Cell and plasma analytes were reported as fold-changes from the mean of Nil mice or “nil normalised”, as described in the methods for both the D7 and D14 time points and presented on a log-scale (with numbers of 0-value data points indicated on the axis). Means and SEM are indicated (shown in yellow) and mean equality was tested using ANOVA on Log (y+0.0001) transformed data using Tukey’s multiple comparisons correction, with 2-way ANOVA and multiple comparison p-values indicated (*, p ≤ 0.05. **, p ≤ 0.01. ***, p ≤ 0.001. ****, p ≤ 0.0001.). Heatmap summaries of the data highlighting the changes are also shown. Three analytes overlapped in the LEGENDplex kits, namely CCL22, CXCL1 and CCL17, and are labelled with a (1) if from the Mac/Mic panel or (2) if they are from the Proinflam panel. (TIFF) [file pone.0264631.s004.tiff]

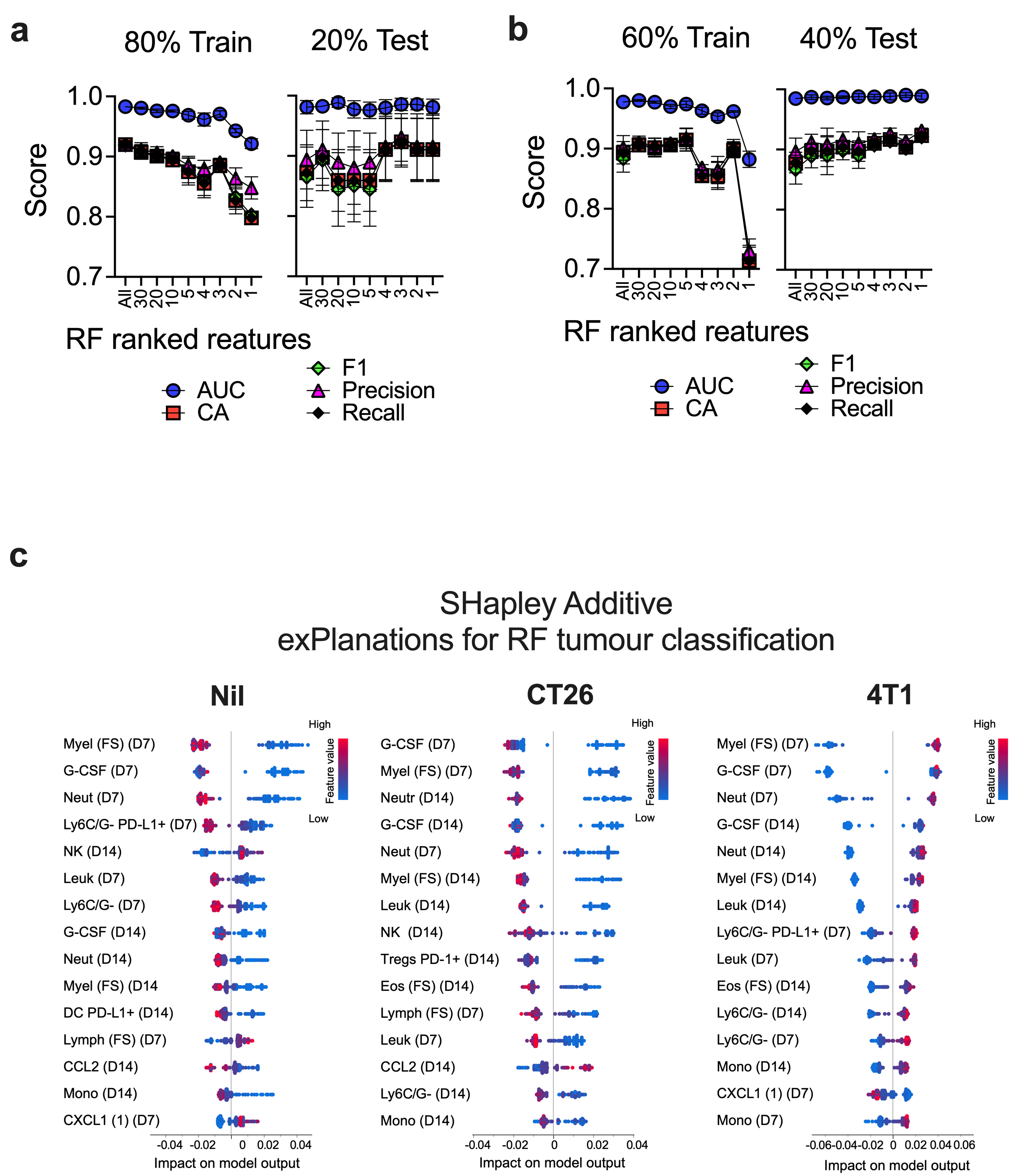

Supplement: S5 Fig — Normalised blood immune features (S4 Fig) taken from the 130 animals that had both D7 and D14 blood samples (Fig 1B), were used in Random Forest modelling to predict presence of tumour and tumour subtype (target classes being Nil, 4T1 and CT26). The model was trained on 80% (a) and 60% (b) of randomly selected data and cross-validated using leave-one-out, and tested using the remaining data. Modelling was done on a progressively smaller number of features, from lowest to highest ranked, based on in-built Random Forest importance for class determination, and the process repeated 3 times. Model performance was assessed by several classification indicators, including area under curve of the receiver operating characteristics (AUC; to assess separability of the classes), classification accuracy (CA; proportion of correct classification), precision (ratio of correct positive prediction to all predicted positive), recall (ratio of correct positive prediction to actual positive), and F1 score (weighted average of precision and recall) with values being from 0 to 1 (and toward the latter being the best). The SHapley Additive exPlanations (SHAP) algorithm feature importance scores for classification using the top-15 features (ranked from highest to lowest) from the SHAP values are shown in (c), and show how the feature values impact on classification of each animal cohort, namely healthily control (Nil), CT26-bearing and 4T1-bearing cohorts. (TIFF) [file pone.0264631.s005.tiff]

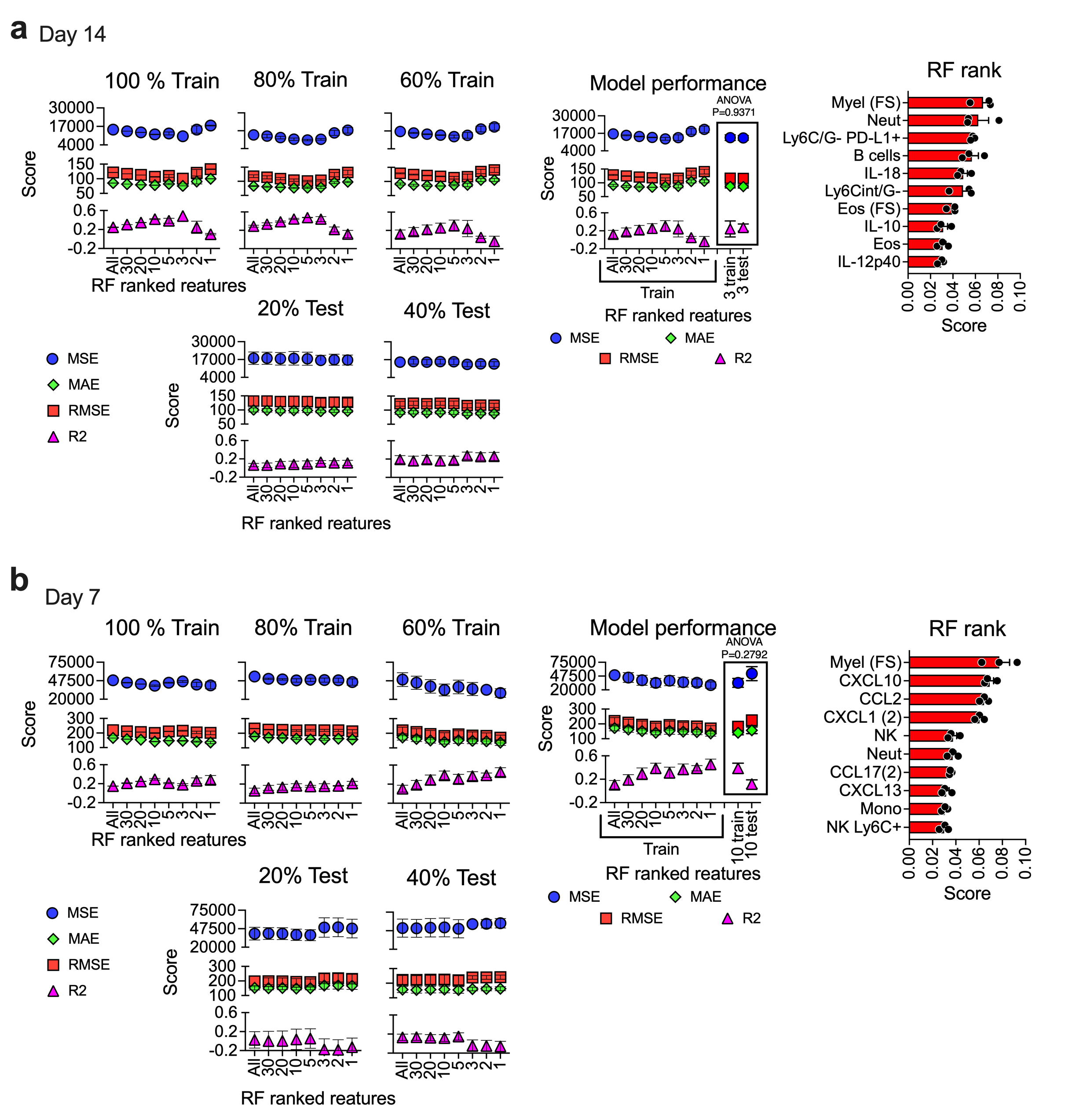

Supplement: S6 Fig — Normalised blood immune features (S4 Fig) taken from 48 CT26-bearing animals that had both D7 and D14 blood samples (Fig 1B) were used in Random Forest modelling to predict CT26 tumour size at D14. The model was trained initially on 100%, 80% and 60% of randomised data and cross-validated using leave-one-out (Train panels) and tested using the remaining data (Test panels). Modelling was done on a progressively smaller number of features, from lowest to highest ranked based on in-built Random Forest importance, and the process repeated 3 times (mean and standard error of mean shown). Model performance was summarised showing the 60%:40%, training:testing split, and equality of test and train performance score means (using the top assigned features) assessed using ANOVA and shown in the main Fig (Fig 3). The Random Forest rank (RF rank) scores for the top-10 features are shown. Model performance was assessed by several regression indicators, including the error scores, Mean Squared Error (MSE), Mean Absolute Error (MAE) and Root Mean Squared Error (RMSE) (which we hoped to minimise), and the coefficient of determination score R2. D14 tumour size was used as the target using D14 blood samples to assess if blood immune features could predict current tumour size (a). D14 tumour size was used as the target using D7 blood samples to assess if blood immune features could predict future tumour size (b). (TIFF) [file pone.0264631.s006.tiff]

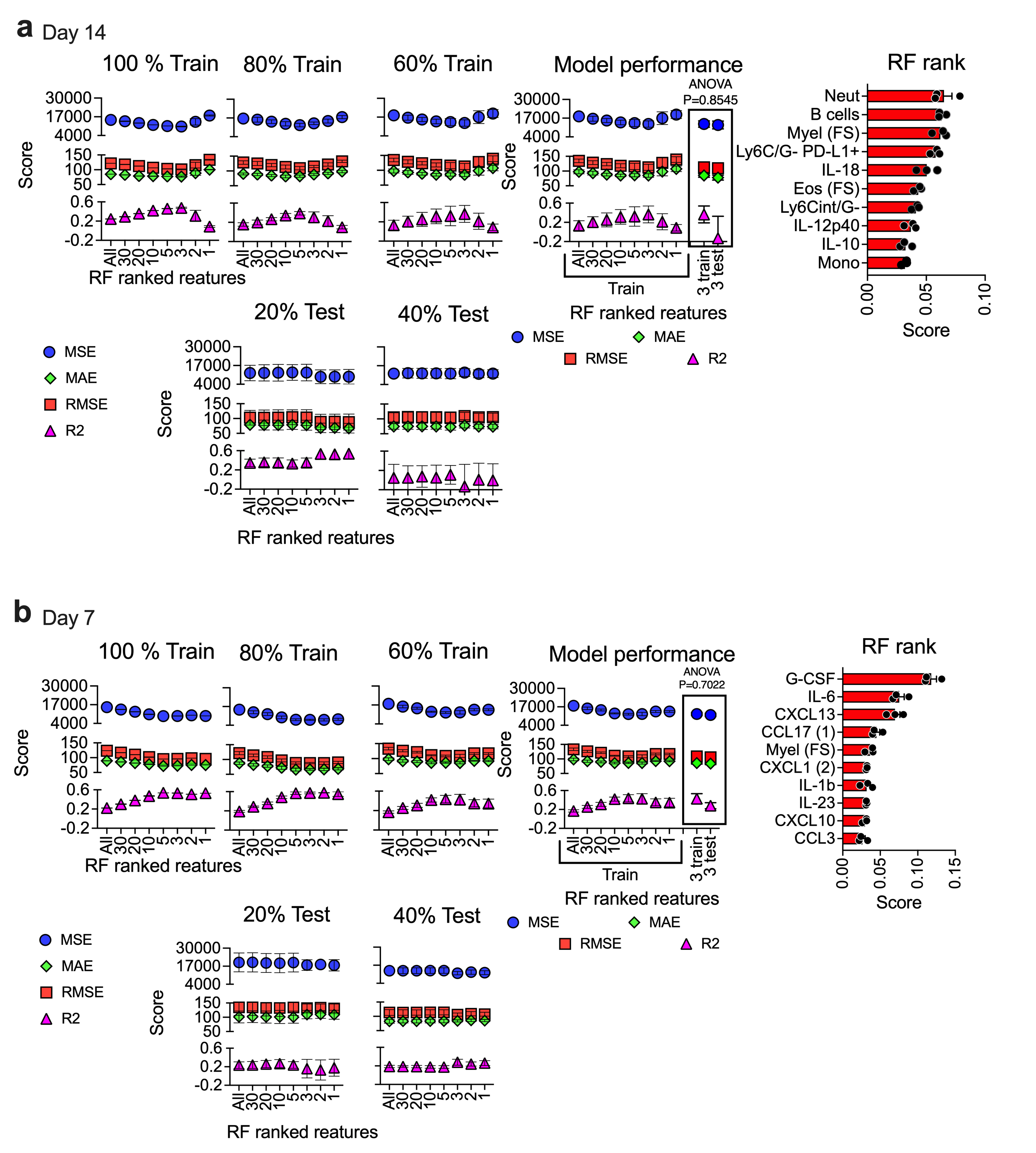

Supplement: S7 Fig — Normalised blood immune features (S4 Fig) taken from 58 4T1-bearing animals that had both D7 and D14 blood samples (Fig 1B), were used in Random Forest modelling to predict 4T1 tumour size at D14. The model was trained initially on 100%, 80% and 60% of randomised data and cross-validated using leave-one-out (Train panels) and tested using the remaining data (Test panels). Modelling was done on a progressively smaller number of features, from lowest to highest ranked based on in-built Random Forest importance, and the process repeated 3 times (mean and standard error of mean shown). Model performance was summarised showing the 60%:40%, training:testing split and equality of test and train performance score means (using the top assigned features) assessed using ANOVA and shown in the main Fig (Fig 4). The Random Forest rank (RF rank) scores for the top-10 features are shown. Model performance was assessed by several regression indicators, including the error scores, MSE, MAE and RMSE (which we hoped to minimise), and the coefficient of determination score R2. D14 tumour size was used as the target using D14 blood samples to assess if blood immune features could predict current tumour size (a). D14 tumour size was used as the target using D7 blood samples to assess if blood immune features could predict future tumour size (b). (TIFF) [file pone.0264631.s007.tiff]
