# Supplementary material for: Machine learning predicts cancer subtypes and progression from blood immune signatures
Source: PLoS One. 2022 Feb 28;17(2):e0264631. doi: 10.1371/journal.pone.0264631 (PMC8884497; doi:10.1371/journal.pone.0264631)
Supplement: S1 Table — (DOCX) [file pone.0264631.s008.docx]

**S1 Table. List of antibodies for cell surface labelling.**

| **Antigen** | **Clone** | **Fluorochrome** | **Cat. #** | **Final dilution** |
| --- | --- | --- | --- | --- |
| CD45 | 30-F11 | PerCP-Cy5.5* | 103132 | 1/200 |
| CD90.2 | 53-2.1 | PE-Cy7* | 105326 | 1/1000 |
| CD4 | RM4-5 | AF-700* | 100536 | 1/400 |
| CD8a | 53-6.7 | FITC* | 100706 | 1/400 |
| PD-1 | 29F.1A12 | APC* | 135210 | 1/200 |
| CD25 | PC61 | APC-F750 | 102054 | 1/200 |
| B220 | RA3-6B2 | AF-700 | 103232 | 1/400 |
| CD11c | N418 | APC | 117310 | 1/200 |
| CD11b | M1/70 | APC-F750* | 101262 | 1/200 |
| Ly6C | HK1.4 | BV-421* | 128032 | 1/800 |
| Ly6G | 1A8 | FITC | 127606 | 1/400 |
| F4/80 | BM8 | PE-Cy7 | 123114 | 1/400 |
| I-A/I-E | M5/114.15.2 | BV-605* | 107639 | 1/400 |
| Siglec-F | S17007L | APC | 155508 | 1/400 |
| CD49b | DX5 | PE* | 108908 | 1/200 |
| NKp46 | 29A1.1 | BV-605 | 331926 | 1/400 |
| PD-L1 | 10F.9G2 | PE-Dazzle594* | 124324 | 1/200 |

*Antibodies used for labelling BD CompBead controls.
