# Supplementary material for: Machine learning predicts cancer subtypes and progression from blood immune signatures
Source: PLoS One. 2022 Feb 28;17(2):e0264631. doi: 10.1371/journal.pone.0264631 (PMC8884497; doi:10.1371/journal.pone.0264631)
Supplement: Attachment — Submitted filename: Response to Reviewers_edits.docx [file pone.0264631.s013.docx]

**Ben J Quah**
Principal Investigator
Irradiation Immunity Interaction Laboratory (I-Cube Lab)
ACRF Department of Cancer Biology & Therapeutics
The John Curtin School of Medical Research
ANU College of Medicine, Biology & Environment
JCSMR Building 131, Garran Road
Acton ACT 2601 Australia
Email: [ben.quah@anu.edu.au](mailto:ben.quah@anu.edu.au)

Academic Editor *PLOS ONE*	24^th^ January, 2022

Dear Editor,

Please find below responses to the specific queries by the reviewers (with reviewers’ statements in blue and our answers to each point in red). We have modified the manuscript’s discussion to address all of these points with track changes.

Reviewer #1: This study is well designed and described.

The major limitations include using (1) cell lines, (2) using only two cell lines, and (3) using mouse xenograft models. The authors allude to (1) in the Discussion (last paragraph), but all of these limitations should be further discussed.

We have now extensively broadened the discussion to include a section (from pg 30) on the use of cell lines and murine cancer models, highlighting their limitations and benefits in cancer research. We have also highlighted the benefits of using more cell line models in this context. It should also be noted that xenografts were not used in this study as suggested by the reviewer, only syngeneic models, and so only syngeneic models were addressed in the new discussion section.

Additionally, the potential translation of this approach to clinical practice should be discussed in greater detail. What are the steps to get there and how would this workflow be applied to actual patients?

We have included details of our clinical approach in the above-mentioned new section of the discussion (from pg 30).

Reviewer #2: This is an excellent study that establish a blood immune signature predicting cancer outcomes by machine learning.

But I still have some advice about this study:

1.It’s better to valid the application of this signature in patients.

We agree very much with this statement and it is indeed what we are currently working towards. The current report was to provide evidence in order to help gain funding and resources to pursue such a human study. Therefore, we feel that to include a human study in this particular report is beyond the scope of the current work. However, we have now included an extensive additional discussion section (as mentioned above from pg 30) highlighting the limitation of our current study and the need for validation in the clinic, and suggesting steps to pursue this.

2.You can add some experiments to certify the result of this study in vivo and in vitro.

While such a general statement is difficult to address specifically, we have modified our discussion section (on pg 26), in which we previously addressed the need for further experimentation to support for our findings, to include a brief detail of the experiments that could be done to support the numerous features we identified that may be involved in cancer development. We note that these experiments would be vast given the number of features involved and are out of the scope of the current study. Instead support our conclusions based on reference to independent peer-reviewed studies that support our findings and which we have summarised in the discussion.

Please note, as requested, we would like to notify the journal that five new references are included in the revised manuscript to help support the changes to the discussion.

Finally, we have taken this opportunity to make some minor amendments to the title, abstract, introduction and discussion, as well as making some minor grammatical changes, which we believe improve the readability of the manuscript without changing the essence of these sections. If this goes beyond what is appropriate, we are happy to exclude these changes and can send this to you instead, please let us know if this is preferred.

Thank you for considering our work for publication in *PLOS ONE.*

Yours sincerely

Dr Ben Quah

Principal Investigator, I-Cube lab

ACRF Department of Cancer Biology & Therapeutics

The John Curtin School of Medical Research

The Australian National University
